# Frequency, associated features, and burden of neurological disorders in older adult inpatients in Brazil: a retrospective cross-sectional study

**DOI:** 10.1186/s12913-017-2260-x

**Published:** 2017-07-24

**Authors:** Aroldo Bacellar, Bruno B. Pedreira, Gersonita Costa, Telma Assis

**Affiliations:** Department of Neurology, Hospital São Rafael, Av. São Rafael 2152, São Marcos, Salvador, BA CEP 41235-190 Brazil

**Keywords:** Nervous system disease, Burden of illness, Length of stay, Patient multimorbidity, Hospital mortality, Patient readmission

## Abstract

**Background:**

The burden of neurological disorders (NDs) in older adult inpatients is often underestimated. We studied diagnostic frequency and comorbidity of NDs among inpatients aged ≥60 years. We compared rates of hospital mortality, length of stay (LOS), and readmission with younger patient counterparts (aged 18–59 years) and older adult non-neurological patients.

**Methods:**

This was a retrospective cross-sectional study of inpatients in a tertiary care center in Brazil. We compiled data for all patients admitted between 1 January 2009 and 31 December 2010, and selected those aged ≥18 years for inclusion in the study. We collected data for inpatients under care of a clinical neurologist who were discharged with primary diagnoses of NDs or underlying acute clinical disorders, and data for complications in clinical or surgical inpatients. Patients who remained hospitalized for more than 9 days were categorized as having long LOS.

**Results:**

Older adult inpatients with NDs (*n* = 798) represented 56% of all neurological inpatients aged ≥18 years (*n* = 1430), and 14% of all geriatric inpatients (*n* = 5587). The mean age of older adult inpatients was 75 ± 9.1 years. Women represented 55% of participants. The most common NDs were cerebrovascular diseases (51%), although multimorbidity was observed. Hospital mortality rate was 18% (95% confidence interval [CI], 15–21) and readmission rate was 31% (95% CI, 28–35), with 40% of patients readmitted 1.8 ± 1.5 times. The long LOS rate was 51% and the median LOS was 9 days (interquartile interval, 1–20 days). In younger inpatients mortality rate was 1.4%, readmission rate was 34%, and long LOS rate was 14%. In older adult non-neurological inpatients, mortality rate was 22%, readmission rate was 49%, and long LOS rate was 30%.

**Conclusions:**

Older adult neurological inpatients had the highest long LOS rate of all patient groups, and a higher mortality rate than neurological patients aged 18–59 years. Readmissions were high in all groups studied, particularly among older adult non-neurological inpatients. Improved structures and concerted efforts are required in hospitals in Brazil to reduce burden of NDs in older adult patients.

## Background

Studies on demographics, prevalence, patterns, and prognosis of neurological disorders (NDs) in older adult inpatients would improve estimates of the burden of illness, healthcare costs, and additional neurology resource needs [1–5]. Globally, epidemiological studies in geriatric populations have shifted from communicable to chronic, noncommunicable diseases [6, 7]. Chronic noncommunicable brain diseases, such as neurodegenerative disorders and stroke are among the leading causes of years of healthy life lost owing to disability [8]. Traumatic brain injury, intracerebral hemorrhage, cerebral ischemia, subarachnoid hemorrhage, and anoxic brain injury are neurological and neurosurgical disorders associated with in-hospital mortality. These disorders account for the highest premature mortality, a public health concern that can be quantified as years of potential life lost [9]. Moreover, nervous system diseases are among the leading contributors of disability-adjusted life years [10, 11]. Although several NDs are often present at death, they might not be reported on death certificates [12]. Health systems are generally configured for individual diseases rather than multimorbidity. This is not appropriate for older adult patients with NDs, who have a high prevalence of multimorbidity; particularly for patients who are economically disadvantaged [13, 14]. Physicians consider NDs difficult to address, and they are often neglected by health authorities and ignored by patients [15, 16]. In addition, although it is well known that stroke and neurological critical care units save lives and can reduce the length of hospital stay, many people with NDs admitted to hospital as emergency cases do not see a neurologist, even in developed countries [17–20].

In developing countries such as Brazil, life expectancy at birth is increasing at a faster rate than that of developed countries. The World Health Organization and the Brazilian Institute of Geography and Statistics reported the life expectancy at birth of Brazilians was 74.6 years in 2011 [21]. By 2040, the geriatric population in Brazil (aged ≥ 60 years) will reach approximately 55 million, which will result in a high prevalence of chronic NDs [22]. Despite increasing hospital admissions of this population, studies on the prevalence of NDs among older adult inpatients are scarce, particularly in Brazil.

This study aimed to record the diagnostic frequency and clinical comorbidity of the most common NDs among older adult inpatients. In addition, as primary outcomes, we compared length of stay, hospital mortality, and readmission rates of older adult neurological inpatients (aged ≥60 years) with those of younger patient counterparts (aged 18–59 years) and with those of older adult non-neurological inpatients.

## Methods

We used an observational, retrospective, cross-sectional, descriptive and analytical design. The study population comprised inpatients who were consecutively admitted to São Rafael Hospital (HSR), a well-equipped, 400-bed general teaching hospital in Salvador, in the state of Bahia, Brazil, between 1 January 2009 and 31 December 2010. The research team comprised 12 clinical neurologists, five neurophysiologists, and four residents in a neurology residency-training program. The hospital neurology department comprises a neurological emergency department with neurologist on duty 7 days/week. In the emergency department, patients with neurological disorders are examined by a neurologist after admission. Triage is performed by a trained nurse. Patients may be referred by other emergency physicians when there is a neurological complication of another illness. The hospital has a neurological ward with a neurohospitalist team that admits and follows neurological patients transferred from the emergency or outpatient departments. Members of this team provide support to other specialists when NDs are comorbid with other diseases. The neurological multidisciplinary team includes physicians, physiotherapists, nurses, pharmacists, nutritionists, speech therapists, and social workers. The team attends a ward round and reviews all medical records each week. HSR is a tertiary medical center fully accredited by the National Organization for Accreditation. It is equipped for CT scans, MRI, and most software necessary for diagnosing acute neurological disorders (including angiography), as well as other facilities for modern neurological care. However, at the time of this study, HSR did not have a neurological intensive care unit or stroke unit.

### Data collection and patient population

We collected data from patients’ electronic medical records for NDs as primary or secondary discharge diagnoses registered using International Classification of Diseases tenth revision (ICD-10) codes. To find all patients with NDs, the hospital information technology section also identified all admissions during the study period and every neurological intervention by searching the billing code used by all neurologists. As a neurologist was on duty in the emergency department, and neurohospitalists provided care for patients in the neurological ward every day (including weekends), we captured most NDs that influenced patient outcomes. After this electronic selection, we carefully reviewed all written medical records for neurological inpatients aged ≥18 years. We selected patients based on specific inclusion criteria: (1) patients with NDs admitted for care to the neurology department; (2) those admitted with clinical complications owing to an underlying ND that required care or therapy by a neurologist; and (3) medical, surgical, or neurosurgical patients with neurological complications during their hospital stay that required consultation with a neurologist. This method captured the majority of NDs and patients with more than one ND. However, we were unable to collect data for patients with more stable NDs who were admitted as having comorbid NDs with other clinical or surgical disorders and did not require neurological consultation during the study period, as NDs were not registered on their discharge summary.

Exclusion criteria were: (1) patients who were admitted by or consulted with a neurologist but had no neurological diagnoses; (2) those whose medical records lacked important data; (3) patients with acute trauma, subarachnoid hemorrhage, central nervous system tumor, or other neurosurgical diseases who were transferred for neurosurgical care; and (4) patients transferred to another hospital without a confirmed diagnosis.

### Neurological disorders

We investigated NDs that commonly affect inpatients: cerebrovascular disorders (ischemic stroke, transient ischemic attack, and spontaneous brain hemorrhage, excluding subarachnoid hemorrhage); epilepsy and acute seizures; movement disorders (including Parkinson’s disease and parkinsonism); neuromuscular disorders; cognitive disorders (including dementia, mild cognitive impairment, and delirium); central nervous system infections; headaches as a main symptom or comorbidity; syncope or near-syncope; toxic and metabolic disorders (including alcoholism); other toxic or metabolic encephalopathy; and acute neurologic complications of hydroelectrolytic disturbances.

Patients with central nervous system neoplastic disorders were those under the care of an oncologist or neurosurgeon who consulted with a neurologist for clinical treatment of NDs, such as epilepsy or cognitive disorders as consequences of brain tumors. Less common NDs that affect older adult patients were grouped as miscellaneous, and included transient global amnesia; dizziness and giddiness (including light-headedness, vertigo, and vertiginous syndromes); hypertensive encephalopathy; and some sleep disorders.

### Clinical comorbidities and patient multimorbidity

We aimed to collect all morbidities in our older adult inpatient population by adopting these criteria:

(i) *Hypertension:* diagnosed according to the Joint National Committee on the Prevention, Detection, Evaluation, and Treatment of High Blood Pressure criteria [23].
(ii) *Dyslipidemias:* defined according to recommendations of the National Cholesterol Education Program Expert Panel on Detection, Evaluation, and Treatment of High Blood Cholesterol in Adults (Adult Treatment Panel III) and the results of recent clinical trials [24, 25].
(iii) *Diabetes mellitus:* diagnosed based on the follow-up report of the 2007 guidelines of the American Diabetes Association [26].
(iv) *Clinical disorders:* classified according to the ICD-10 and including: a) infections, represented not only by patients who were admitted with infections but also neurological patients who experienced infections during their hospitalization; b) neoplasms; c) chronic and acute respiratory system diseases; d) musculoskeletal diseases; e) genitourinary disorders; f) digestive, endocrine, and metabolic disorders, including water and electrolyte disturbances (hepatic insufficiency was considered a metabolic rather than a digestive disorder); and g) circulatory system disorders (cardiac and peripheral vascular disorders), excluding patients with stroke.

### Hospital mortality, lengths of hospital stay, and patient readmission

We computed rates of hospital mortality, readmission, and LOS in older adult non-neurological inpatients and patients aged 18–59 years admitted with NDs during the study period. This allowed comparisons of the findings in these two populations against the target population of older adult neurological inpatients. The median LOS for the entire older adult population was 9 days. Therefore, we categorized patients who remained hospitalized for more than 9 days as having long LOS. We recorded all hospital readmissions during the 2-year study period.

### Ethics

This study was approved by the HSR Ethical Committee for Research (number 8/11) on 25 August, 2011. The HSR Ethical Committee for Research was accredited by the National Committee for Ethics in Research, according to the Operational Manual for Ethics Committee in Research [27].

### Statistical analysis

Normally distributed numerical variables are presented as means and standard deviations. Non-normally distributed variables are presented as medians and interquartile intervals. Categorical variables are reported as proportions, and we defined confidence intervals (CI) at 95%. Variables were compared using Pearson’s chi-squared tests. Statistical significance was determined at a *p*-value of ≤0.05. SPSS Version 21 (IBM Corp., Armonk, NY, USA; licensed by DMSS Software Ltda., Sao Paulo, Brazil) was used for data analyses.

## Results

### Patient demographics

**Fig. 1:**
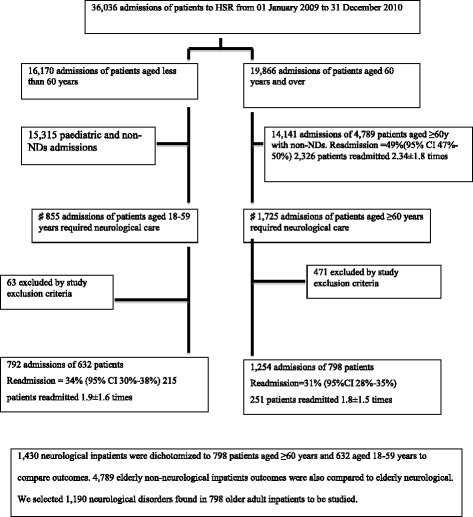
Patient selection flow-chart. NDs, neurological disorders ♯The authors reviewed the medical records of each of the 1725 admissions after the electronic selection performed by Information Technology section of HSR

In total, 1430 inpatients aged ≥18 years who met our selection criteria were admitted to HSR with NDs during the study period. Of these, 798 (56%) were older adult inpatients, representing 14% of the total geriatric inpatient population (5587 patients) (Fig. 1).

The average age of older adult neurological inpatients was 75.7 ± 9.1 years, and the median age was 76 years. Overall, 25% of the study population was aged 82 years and above, and 55% were women.

### Diagnostic frequency and patient multimorbidity

Among our older adult inpatient population, 555 (70%) had primary NDs under the care of a neurologist listed (ICD-10) in their discharge summaries. In addition, 243 (30%) patients were admitted with clinical complications of underlying chronic NDs requiring neurological care, or were clinical or surgical patients with neurological complications. The latter were obtained by searching the neurologists’ billing code. Overall, 350 (44%) patients were affected by more than one ND, accounting for 392 additional NDs that were classified as neurological comorbidities in this population. Therefore, there were 1190 ND diagnoses in 798 older adult inpatients, representing a mean patient neurological multimorbidity of 1.49 (95% CI, 1.42–1.56), with a range of 1–5 NDs per patient. The diagnostic frequencies of NDs in older adult inpatients, distributed by sex, are shown in Table 1.

**Table 1:** Diagnostic frequency of neurological disorders among older adult inpatients, distributed by sex

Neurological disorder	Men (*n* = 363)	Women (*n* = 435)	Total (*n* = 798)
Cerebrovascular disease	186 (51%)	219 (50%)	405 (51%)
Cognitive disorder	97 (27%)	102 (23%)	199 (25%)
Movement disorder	75 (21%)	65 (15%)	140 (17%)
Epilepsy	62 (17%)	65 (15%)	127 (16%)
Syncope	53 (15%)	69 (16%)	122 (15%)
Headache	42 (12%)	60 (14%)	102 (13%)
Neuromuscular	19 (5%)	36 (8%)	55 (7%)
Toxic & metabolic	7 (2%)	5 (1%)	12 (1%)
CNS infection	3 (0.8%)	2 (0.5%)	5 (0.6%)
Brain injury	0	3 (0.7%)	3 (0.4%)
CNS neoplastic	2 (0.6%)	0	2 (0.3%)
Miscellany	8 (2%)	10 (2%)	18 (2%)
Total	554	636	1190

*Abbreviation*: *NDs* neurological disorders, *CNS* central nervous system

Note: A total of 350 patients (40%) had more than one neurological disorder (patient neurological multimorbidity)

Although it was not our purpose to compare the patterns of neurological disorders between younger and older cohorts, it is worth mentioning that these patterns were diverse. In the study period, 33% of the patients aged 18–59 years were admitted for headache, 17% had cerebrovascular disorders, 8% had epilepsy and seizures, and 8% had neuromuscular disorders. There were also other diseases not reported in the older adult inpatients in our sample such as multiple sclerosis and other demyelinating disorders (10%). Infections were higher in the younger cohort (6%) and only a small number of patients were admitted with cognitive disorders (2%) or movement disorders (2%). Disorders grouped as miscellaneous were present in 8% of the admitted patients in our population, and other NDs each accounted for less than 1% of patients.

### Clinical comorbidities

We observed a remarkable number of comorbidities in our older adult inpatient population, the most common being hypertension (85%) and diabetes mellitus (58%). We also found multimorbidity in this patient group, with a mean of 3.2 ± 1.47 (95% CI, 3.1–3.3), a median of 3, and a range of 1–8 comorbidities per patient. The total number of comorbidities and their frequency distributed by sex are shown in Table 2.

**Table 2:** Frequency of comorbidities in older adult inpatients admitted with NDs, by sex

Comorbidity	Men (*n* = 363)	Women (*n* = 435)	Total (*n* = 798)
Hypertension	300 (83%)	374 (86%)	674 (85%)
Diabetes mellitus	213 (59%)	246 (57%)	459 (58%)
Dyslipidemia	153 (42%)	210 (48%)	363 (46%)
Infection	167 (46%)	192 (44%)	359 (45%)
Neoplasm	93 (26%)	88 (20%)	181 (23%)
Endocrine & metabolic	52 (14%)	70 (16%)	122 (15%)
Circulatory disorder	53 (15%)	49 (11%)	102 (13%)
Urogenital system	46 (13%)	46 (11%)	92 (12%)
Respiratory disorder	45 (12%)	41 (9%)	86 (11%)
Musculoskeletal disease	31 (9%)	55 (13%)	86 (11%)
Digestive system	28 (8%)	29 (7%)	57 (7%)
Total	1181	1400	2581

*Abbreviation*: *NDs* neurological disorders

Note: Patient multimorbidity rate was 88%, and 343 (43%) had four or more comorbidities

### Length of stay, hospital mortality, and readmission rates

**Fig. 2:**
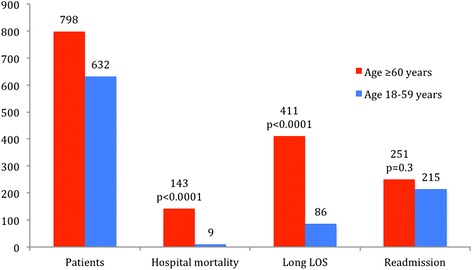
Frequency, hospital mortality, long LOS, and readmission, according to age group, in patients admitted with NDs. Abbreviations: LOS, length of stay; NDs, neurological disorders

**Fig. 3:**
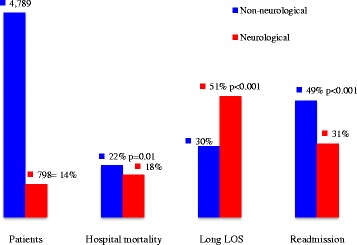
Rates of frequency, hospital mortality, long LOS, and readmission in older adult inpatients admitted to HSR between 1 January 2009 and 31 December 2010. Abbreviations: LOS, length of stay; HSR, São Rafael Hospital

The median LOS for older adult neurological inpatients was 9 days, with an interquartile interval of 1–20 days; 411 (51%) of these patients had LOS of ≥9 days (95% CI, 48–55). The hospital mortality rate was 18% (95% CI, 15–21). Over the 2-year study period, 251 patients were readmitted, giving a 31% readmission rate (95% CI, 28–35). Of these patients, 40% were readmitted more than once, giving an average of 1.8 ± 1.5 readmissions. We compared these data with those of neurological patients aged 18–59 years and with older adult non-neurological inpatients. We found that older adult inpatients admitted with NDs had the highest rates of long LOS among the three populations and much higher rates of mortality than their younger counterparts. However, they had lower mortality rates than older adult non-neurological inpatients. Readmission rates for all three populations were high, but particularly among older adult non-neurological inpatients (Figs. 1, 2 and 3).

## Discussion

Globally, there are few studies covering nervous system diseases among inpatients. The majority of published studies are from African countries; they include all age groups and report more acute NDs, including central nervous system infections [1, 2]. It was not the purpose of this study to define the prevalence of NDs among all inpatients. We aimed to investigate the frequency and burden of all acute and chronic NDs and comorbidities, with a focus on multimorbidity among older adult inpatients attended by neurologists. The study method we used to capture NDs has been used in other ND inpatient surveys [28, 29]. As HSR has a neurologist physically present in the emergency department at all times and neurohospitalists on the ward, our team examined almost all neurological inpatients. HSR has a very organized neurological service, which is an exception in Brazil. Neurological consultation in the emergency department is associated with better care, fewer unnecessary admissions, and lower costs [29]. Neurohospitalists are also important, and associated with better patient management [30, 31]. Therefore, we assumed that the ND outcome scenario is generally worse in the majority of Brazilian hospitals. As mentioned, HSR does not have a stroke unit or neurological critical care unit. Such facilities are lacking in Brazil, even at university teaching hospitals, and particularly in the northeast region. Our study population was older, with median age of 76 years and 25% were aged ≥82 years. We found that older adult neurological inpatients were more numerous than their younger counterparts. There were many reasons for the diversity of outcomes between the studied populations, but ageing is a key issue of concern.

Cerebrovascular disorders were the most prevalent diseases in the older adult study population, and these are known to be related to higher rates of readmission, longer LOS, higher mortality, and greater costs [32]. We also observed many other NDs such as epilepsy, seizures and status epilepticus, dementia, Parkinson’s disease, and chronic conditions in patients frequently admitted as having underlying NDs. Neuromuscular disorders, including diabetic neuropathies, motor neuron disease, polyneuropathy, and critical illness myopathy were also seen in our geriatric population.

There were situations in which a ND was a complication of another underlying chronic ND, such as epilepsy in Alzheimer-type dementia or cerebrovascular diseases. We found that 44% of older adult inpatients had had two or more NDs. Our study revealed a significant difference in the total number of the most common NDs reported at administrative discharge: 555 (46%) as primary diagnoses compared with the total (1190) obtained after careful scrutiny of the medical records. This “neurological multimorbidity” is important as it is frequently neglected in medical statistics, which usually only register primary diseases listed in discharge summaries [9, 13]. In our study, older adult neurological inpatients accounted for a large number of critical patients admitted with cerebrovascular disorders, seizures, status epilepticus, severe infections complicating chronic nervous system disorders, and patients with multimorbidity. This is a matter of concern because dementia can increase severe sepsis and the mortality rate of inpatients admitted for other diseases [33]. As stroke units and neurological critical care units are associated with better outcomes and shorter LOS in these patients, our study highlights the need for specialized neurological units to adequately address this issue.

The geriatric population in this study had a large number of clinical comorbidities (88% with two or more), with a mean multimorbidity of 3.2 ± 1.3 and a median of three comorbidities per patient. Hypertension and diabetes were the most prevalent comorbidities, which might explain the large number of cerebrovascular disorders. We also observed other serious diseases including neoplasms, chronic renal failure, atrial fibrillation, and acute comorbidities (e.g., infections and severe sepsis). Previous studies have shown that clinical multimorbidity affects 99% of older adult women (≥65 years) and 97% of men [34], and these comorbidities increase the risks for adverse outcomes and the cost of healthcare [14, 35]. The mortality rate for older adult neurological inpatients (18%) was lower than that reported in African neurological inpatients, perhaps because those studies included more participants with severe infections such as HIV and brain tumors [1, 2]. However, compared with neurological inpatients aged 18–59 years, mortality among older adult patients was more frequent and there were differences in mortality and long LOS rates.

Brazil’s population is aging rapidly, and it is essential that hospitals are prepared to meet the challenge of the country’s changing demographics. The hospital LOS in geriatric populations is normally longer than that of younger patients owing to the complexity and severity of the case mix, including functional status, cognitive score, poor nutrition, multimorbidity, illness severity, and polypharmacy [36]. Moreover, our study suggests that older adult neurological inpatients have an even greater likelihood of longer LOS than other geriatric populations. The patient readmission rate was 31%; 40% of these cases were readmitted more than once, resulting in a large number of readmissions. This was similar to the readmission rate among patients aged 18–59 years, but lower than that for older adult non-neurological inpatients. However, it should be noted that the study population included very old patients, who are typically more likely to be readmitted [37]. Readmissions in older adult populations are common; our rate was similar to that found in a larger population [38]. This demonstrates that surgical patients might have greater likelihood of readmission, which could explain why older adult non-neurological inpatients had higher rates of readmission [38]. Readmission occurs for several reasons, and usually in patients with long LOS; however, nearly half of readmissions are avoidable [39]. Actions to improve patient discharge processes and to promote neurological monitoring of patients after discharge may help prevent rehospitalization [40].

Some limitations of this study must be noted. As stated, it was not our intention to define the real prevalence of NDs in this survey, as we did not evaluate patients with NDs admitted to the neurosurgical department. In addition, patients with stable NDs were not captured if they did not require a neurological consultation. However, the majority of studies on neurological inpatients (which are the most referenced in prospective surveys of epidemiological multimorbidity) did not include patients with stable NDs as comorbidities [41]. In addition, retrospective inpatient registries might have selection bias owing to differences in admission criteria, number of hospital beds, or population characteristics.

## Conclusions

We found nervous system disorders, clinical disorders with neurological underpinnings, neurological complications of systemic disorders, and multimorbidity were common in our older adult inpatient population, and exceeded those found in younger patient counterparts. Although it was not possible to capture stable NDs as comorbidities, we were able to evaluate all NDs that influenced patient outcomes as well as those registered as primary diagnoses. We found higher hospital mortality and longer LOS rates among older adult neurological inpatients than among younger patients with NDs. The rate of long LOS was also higher in older adult neurological inpatients than in older adult non-neurological inpatients. These factors impose a substantial burden on the patient, and immense costs to the community and healthcare system. Brazil’s growing older adult population is likely to bear an even greater ND-related burden in the coming years. This is a concerning public health problem, and efforts must be made to structure hospitals to adequately address this challenge. Our finding that readmissions were high in all inpatient populations presents another cause for concern.

## Abbreviations

CONEP: The National Committee for Ethics in Research (Brazilian)
HSR: Hospital Sao Rafael
ITS: Information Technology Section
LOS: Length of stay
NDs: Neurological disorders
